# Imputation of high-density genotypes in the Fleckvieh cattle population

**DOI:** 10.1186/1297-9686-45-3

**Published:** 2013-02-13

**Authors:** Hubert Pausch, Bernhard Aigner, Reiner Emmerling, Christian Edel, Kay-Uwe Götz, Ruedi Fries

**Affiliations:** 1 Lehrstuhl fuer Tierzucht, Technische Universitaet Muenchen, 85354, Freising, Germany; 2 Institut fuer Tierzucht, Bayerische Landesanstalt für Landwirtschaft, 85586, Poing, Germany

## Abstract

**Background:**

Currently, genome-wide evaluation of cattle populations is based on SNP-genotyping using ~ 54 000 SNP. Increasing the number of markers might improve genomic predictions and power of genome-wide association studies. Imputation of genotypes makes it possible to extrapolate genotypes from lower to higher density arrays based on a representative reference sample for which genotypes are obtained at higher density.

**Methods:**

Genotypes using 639 214 SNP were available for 797 bulls of the Fleckvieh cattle breed. The data set was divided into a reference and a validation population. Genotypes for all SNP except those included in the BovineSNP50 Bead chip were masked and subsequently imputed for animals of the validation population. Imputation of genotypes was performed with *Beagle*, *findhap*.*f90*, *MaCH* and *Minimac*. The accuracy of the imputed genotypes was assessed for four different scenarios including 50, 100, 200 and 400 animals as reference population. The reference animals were selected to account for 78.03%, 89.21%, 97.47% and > 99% of the gene pool of the genotyped population, respectively.

**Results:**

Imputation accuracy increased as the number of animals and relatives in the reference population increased. Population-based algorithms provided highly reliable imputation of genotypes, even for scenarios with 50 and 100 reference animals only. Using *MaCH* and *Minimac*, the correlation between true and imputed genotypes was > 0.975 with 100 reference animals only. Pre-phasing the genotypes of both the reference and validation populations not only provided highly accurate imputed genotypes but was also computationally efficient. Genome-wide analysis of imputation accuracy led to the identification of many misplaced SNP.

**Conclusions:**

Genotyping key animals at high density and subsequent population-based genotype imputation yield high imputation accuracy. Pre-phasing the genotypes of the reference and validation populations is computationally efficient and results in high imputation accuracy, even when the reference population is small.

## Background

With the availability of dense marker panels, assessing the genetic value of individuals without relying on phenotypic information is possible [1]. Current routine genomic evaluation of cattle populations is performed using the genotypes of ~54 000 SNP. However, the most recent high-density genotyping arrays facilitate the high-throughput interrogation of 648 874 and 777 962 SNP, respectively [2]. Using densely spaced marker maps increases the probability of co-segregation of SNP and quantitative trait nucleotides (QTN) [3]. Since both genomic predictions and genome-wide association studies exploit linkage disequilibrium (LD) between anonymous markers and QTN, increasing the density of SNP maps is likely to improve the capacities of genome-wide population analyses [4-9]. However, the relationship between validation and calibration populations is crucial to obtain accurate genomic predictions [10].

Genotype imputation is invaluable to combine different marker panels and to infer missing genotypes [11]. Imputation of genotypes makes it possible to extrapolate genotypes from lower to higher density arrays based on a representative sample of individuals genotyped at high-density. Different approaches for imputation of genotypes exploit pedigree information [12], population-wide LD (*e*.*g*. [13,14]) or both sources of information (*e*.*g*. [15]).

The accuracy of genotype imputation depends on the proportion of missing genotypes [16] and the number of individuals and relatives genotyped at high-density [17,18]. However, the number of reference genotypes required to ensure high imputation quality varies across populations and depends mainly on the effective population size [19]. Careful selection of animals for high-density genotyping facilitates population-wide imputation of high-quality genotypes while minimizing genotyping costs [20,21].

Here, we report the evaluation of four tools for imputation of genotypes in 797 Fleckvieh (FV) bulls genotyped with 639 214 SNP. We show that imputation based on pre-phasing results in high accuracy and is computationally efficient. As few as 100 informative reference animals were sufficient to genotype the entire population with high accuracy.

## Methods

### Animals

A total of 814 bulls of the FV bovine breed were genotyped with the Illumina BovineHD Bead chip including genotypes of 777 962 SNP. The animals were born between 1970 and 2007 with 90.2% born between 1997 and 2004 (see Additional file 1). The bulls descended from 209 sires and 223 maternal grand-sires. The paternal half-sib and maternal grand-sire families comprised up to 27 and 46 members with an average of 3.9 and 3.2 members, respectively.

### Genotypes and quality control

Genotype calling was performed using the default parameters of Illumina's *BeadStudio*. The chromosomal position of the SNP was determined based on the UMD3.1 assembly of the bovine genome [22]. We excluded 1224 Y-chromosome, 343 mitochondrial and 1735 SNP with an unknown chromosomal position from further analysis. One SNP out of 55 pairs of SNP with identical chromosomal positions but different SNP-ids (duplicates) was omitted. Eight bulls were excluded because genotyping failed for more than 5% of the SNP. We omitted 10 751 SNP because genotyping failed in more than 5% of the individuals, 124 652 SNP that had a minor allele frequency (MAF) < 1% and 4024 SNP with a significant (P < 10^-6^) deviation from the Hardy-Weinberg equilibrium. Pedigree-based relationships among the animals were obtained using *PyPedal*[23], tracing pedigree information back to 1920. Comparing the pedigree and the realized genomic relationships [24] led to the exclusion of nine animals showing major discrepancies (see Additional file 2). Genotypes of 228 sire-offspring pairs were inspected for mendelian errors (*e*.*g*. genotype AA and BB in sire and offspring, respectively). The number of mendelian errors ranged from 24 to 132, with an average of 63 errors per pair. Genotypes of both sire and offspring were set to missing for SNP with mendelian errors. The final data set comprised 797 animals and 639 214 SNP, with an average call-rate of 99.48% per individual.

### Evaluation of imputation accuracy

The high-density data set was divided into a reference and a validation population. Complete genotype information was retained for animals in the reference population, whereas genotypes were set to missing for animals in the validation population for all SNP except those included in the BovineSNP50 Bead chip (version 2). SNP that are present in the BovineSNP50 Bead chip but not in the BovineHD Bead chip were not considered. Subsequently, genotype imputation was performed to infer the masked genotypes *in silico*. Imputation accuracy was assessed by comparing the imputed genotypes/alleles with the true genotypes/alleles and by calculating the correlation between true and imputed genotypes (r_TG,IG_) [25]. The SNP-specific imputation accuracy was assessed as a function of allele frequencies. Allele frequencies and the corresponding proportion of correctly imputed alleles were fitted with a local regression model (LOESS), with a smoothing factor of 0.1. The individual-specific imputation accuracy was calculated as a function of the number of relatives in the reference population. The relationship was obtained from the pedigree-based numerator relationship matrix (see above).

### Selection of reference animals

Imputation accuracy was evaluated for four scenarios with an increasing number of reference animals. Animals for the reference population were selected based on $p_{\text{m}}=A_{\text{m}}^{-1}c_{\text{m}}$[20], where **A**_m_ is a subset of the numerator relationship matrix, **c** is a vector representing the average relationship of m selected animals with the entire population and **p** is a vector of the proportion of the gene pool captured by the m animals. Out of 797 animals with high-density genotypes, the most informative 50, 100, 200 and 400 animals were iteratively chosen to maximise $\sum_{i=1}^{m}p_{i}$, *i*.*e*. the most informative 50 animals were a subset of the most informative 100 animals *etc*. Such subsets of animals capture the greatest proportion of the gene pool of the entire dataset and should provide the most accurate genotype imputation. Animals identified in this way were considered as reference individuals. The remaining 747, 697, 597 and 397 animals were used as validation individuals. Imputation accuracy was also assessed using randomly selected reference animals. Fifty animals were randomly selected as reference individuals and the remaining 747 animals were used as validation individuals. The random selection of reference animals and subsequent genotype imputation and validation were repeated ten times.

### Imputation algorithms

The performance of four imputation tools was evaluated. Three population-based imputation algorithms (*Beagle* (version 3.2.1) [13], *MaCH* (version 1.0.16.a) [14], *Minimac*[26]) exploiting LD were applied without considering pedigree information. Additionally, the performance of an algorithm based on long-range phasing implemented in *findhap*.*f90* (version 2) [6], combining both family and population-based imputation, was evaluated. *Beagle* and *MaCH* were applied since these algorithms provide high imputation accuracy in both livestock and human populations [27,28]. *Beagle* and *MaCH* are imputation algorithms based on a hidden Markov model (HMM). *Beagle* performs a local clustering of haplotypes at each marker position to define the hidden states whereas *MaCH* samples pairs of known haplotypes for each individual based on the observed genotypes in each round of the HMM. A detailed review of the implemented algorithms is given in [29]. *MaCH* is time consuming, especially for large reference populations [30]. Thus, we also evaluated *Minimac*, a computationally efficient “pre-phasing”-based implementation of the *MaCH* algorithm, taking haplotypes as input for both the reference and the validation populations. Therefore, haplotypes for the reference and validation populations were inferred using *Beagle* and subsequent haplotype-based imputation of missing genotypes was performed with *Minimac*. While *Beagle*, *MaCH* and *Minimac* provide allele dosage data (*i*.*e*. continuously distributed values ranging from 0 to 2), *findhap*.*f90* provides discrete genotypes only (*i*.*e*. 0,1,2). For the present study, discrete genotypes were analysed and missing alleles resulting from imputation with *findhap*.*f90* were subsequently imputed based on allele frequencies. All programs were run on an Intel Xeon 2.13 Ghz processor using recommended parameters. A detailed overview of the parameters applied with the different tools is given in Additional file 3.

### Identification of misplaced SNP

Genome-wide analysis of imputation accuracy detected regions of poor imputation quality, most likely because of misplacement of SNP. The chromosomes were partitioned into segments of 0.5 Mb. The segments were inspected for SNP with more than 10% incorrectly imputed genotypes. If more than ten SNP with more than 10% of incorrectly imputed genotypes were located within a segment, all the SNP within this segment were considered as misplaced. If less than ten SNP per segment had more than 10% incorrectly imputed genotypes, only the erroneously imputed SNP were considered as misplaced.

## Results

Genotypes for animals of the validation population were imputed based on an increasing number of highly informative reference animals with high-density genotypes. 78.03% of the genes/haplotypes of the 797 studied animals could be traced back to the subset of the 50 most informative reference animals. This fraction increased to 89.21%, 97.47% and > 99% for the scenarios including 100, 200 and 400 reference animals. Most of the 797 animals (90.2%) were born between 1997 and 2004 (see Additional file 1) and the number of sire-offspring pairs was low (n = 228). Within the subset of the 50 most informative animals, the majority (56%) was born before 1997. These animals can be considered as the ancestors of the studied population. Most validation animals had no first-degree relatives in the reference population (Table 1). The fraction of validation animals without relatives with a pedigree relationship greater than 0.25 was 18.07% (135 of 747) and 3.78% (15 of 397) in the scenarios including 50 and 400 reference animals, respectively. However, the average number of related reference animals was very small for the validation animals across all scenarios (Table 2). Imputation of genotypes was performed separately for six chromosomes (Table 3). The number of masked and subsequently imputed high-density genotypes ranged from 93.44% (BTA1) to 94.41% (BTA5). The validation populations contained no missing genotypes after imputation with *Beagle*, *MaCH* and *Minimac*. However, 6.49%, 1.46%, 0.26% and 0.11% of the masked genotypes remained missing after imputation with *findhap*.*f90* for the scenarios including 50, 100, 200 and 400 reference animals, respectively. Those genotypes were subsequently imputed based on allele frequencies.

**Table 1:** Number of validation animals without close relatives in the reference population

	**Scenario (number of reference animals / number of validation animals)**			
	**50 / 747**	**100 / 697**	**200 / 597**	**400 / 397**
no relatives with r ≥ 0.50	621	562	453	316
no relatives with r ≥ 0.25	135	62	30	15
no relatives with r ≥ 0.125	16	4	-	-
no relatives with r ≥ 0.0625	5	2	-	-

The number of validation animals without close relatives in the reference population is presented for four different classes of relationship (r) and four scenarios with an increasing number of reference animals. Since most animals in our study were born between 1997 and 2004, the number of validation animals without close relatives in the reference population was very high across all scenarios.

**Table 2:** Average number of relatives in the reference population

**Scenario (reference animals / validation animals)**	**Average number of relatives in the reference population**			
	**r ≥ 0.50**	**0.25 ≥ r < 0.50**	**0.125 ≥ r < 0.25**	**0.0625 ≥ r < 0.125**
50 / 747	0.18	1.15	1.86	4.57
100 / 697	0.21	1.61	2.43	8.64
200 / 597	0.27	2.51	4.16	20.89
400 / 397	0.27	4.82	9.60	54.58

The average number of relatives in the reference population is given for the animals in the validation population for four classes of relationships (r) and four scenarios with an increasing number of reference animals. The average number of close relatives in the reference population was very small for most animals in the validation population.

**Table 3:** Number of SNP used for the evaluation of imputation accuracy on six chromosomes

**Chr**	**Chromosome-length [Mb]**	**Number of high-density SNP in the reference population**	**Average distance between two high-density SNP [bp]**	**Number of medium-density SNP in the validation population**	**Average distance between two medium-density SNP [bp]**
1	158.32	39 167	4042	2568	61 587
5	121.18	29 050	4171	1621	74 740
10	104.30	26 695	3906	1646	62 724
15	85.27	21 425	3978	1280	65 850
20	71.98	19 111	3764	1183	60 530
25	42.85	11 725	3648	744	57 533

Number of high-density SNP passing stringent quality parameters for the six evaluated chromosomes. The medium-density SNP are a subset of the bovineHD BeadChip collection that are interrogated with the BovineSNP50 BeadChip (version 2). SNP positions were determined based on the UMD3.1 assembly of the bovine genome.

### Genotypic imputation accuracy

**Figure 1:**
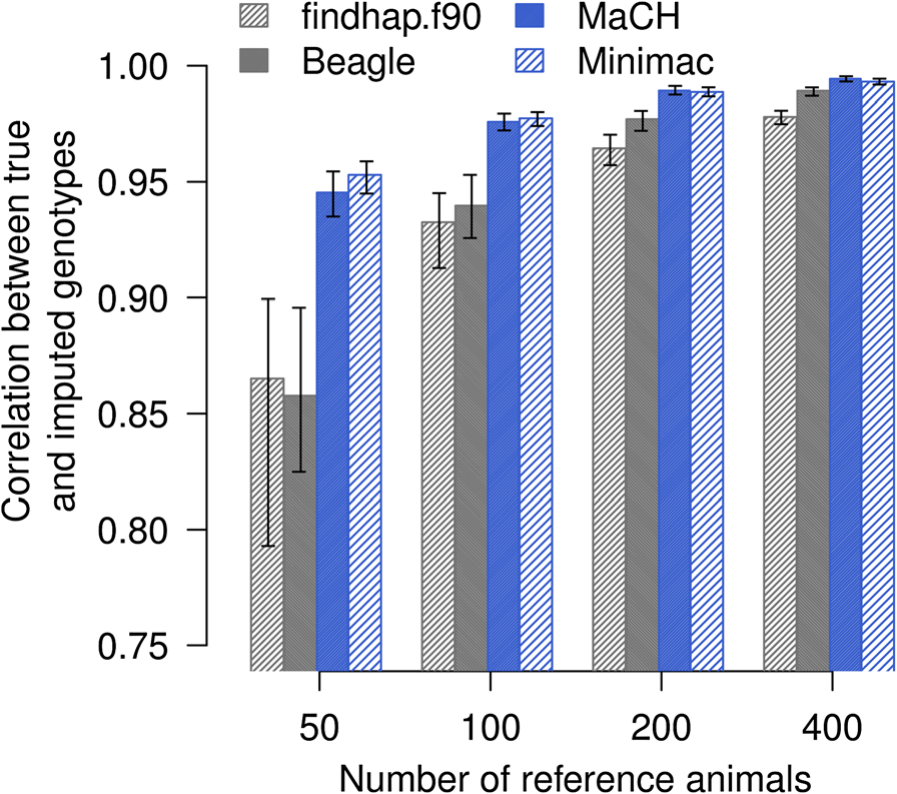
**Imputation accuracy.** Barplots indicate the correlation between true and imputed genotypes (r_TG,IG_) averaged over six chromosomes for an increasing reference population size. The black lines represent the minimum and maximum imputation accuracy for the six chromosomes.

The imputation accuracy increased as the size of the reference population increased (Figure 1). However, the correlation between true and imputed genotypes (r_TG,IG_) varied considerably across chromosomes, especially for the scenario including 50 reference animals. With *Beagle*, the correlation ranged from 0.825 (BTA25) to 0.896 (BTA1) and with *findhap*.*f90* it ranged from 0.793 (BTA25) to 0.899 (BTA2). *MaCH* and *Minimac* provided nearly identical imputation quality, although computational time was considerably lower with *Minimac* (Table 4). Phasing both the reference and validation populations with *Beagle* and subsequent haplotype-based genotype imputation with *Minimac* outperformed all other approaches, especially when the number of reference animals was small. Imputation with *Minimac* yielded an r_TG,IG_ value of 0.953 with 50 reference animals, while with *MaCH*, *Beagle* and *findhap*.*f90* r_TG,IG_ was only 0.945, 0.858 and 0.865, respectively (Table 5). Increasing the number of reference animals to 100, 200 and 400 yielded an r_TG,IG_ value of 0.977, 0.989 and 0.993 with *Minimac*.

**Table 4:** Computing time for the imputation of high-density SNP on chromosomes 1, 15 and 25

**Number of animals in reference and validation population**	**Chr**	***Beagle***	***MaCH***^**a**^	***findhap*.*f90***^**b**^	***Minimac***^**c**^
50 / 747	BTA1	2.67 h	1.30 h (0.03 h / 0.30 h / 0.97 h)	0.07 h	0.17 h (0.03 h / 0.07 h / 0.07 h)
	BTA15	1.18 h	0.68 h (0.02 h / 0.14 h / 0.52 h)	0.04 h	0.09 h (0.02 h / 0.04 h / 0.03 h)
	BTA25	0.67 h	0.37 h (0.01 h / 0.08 h / 0.28 h)	0.04 h	0.05 h (0.01 h / 0.02 h / 0.02 h)
100 / 697	BTA1	3.93 h	5.01 h (0.08 h / 1.11 h / 3.82 h)	0.07 h	0.27 h (0.08 h / 0.06 h / 0.13 h)
	BTA15	2.48 h	2.72 h (0.05 h / 0.55 h / 2.12 h)	0.05 h	0.15 h (0.05 h / 0.03 h / 0.07 h)
	BTA25	1.33 h	1.48 h (0.03 h / 0.32 h / 1.13 h)	0.04 h	0.09 h (0.03 h / 0.02 h / 0.04 h)
200 / 597	BTA1	4.49 h	18.92 h (0.20 h / 4.31 h / 14.41 h)	0.07 h	0.48 h (0.20 h / 0.05 h / 0.23 h)
	BTA15	2.87 h	10.06 h (0.11 h / 2.22 h / 7.73 h)	0.05 h	0.27 h (0.11 h / 0.03 h / 0.13 h)
	BTA25	1.38 h	5.76 h (0.06 h / 1.24 h / 4.45 h)	0.04 h	0.14 h (0.06 h / 0.01 h / 0.07 h)
400 / 397	BTA1	3.73 h	81.23 h (0.44 h / 21.97 h / 58.82 h)	0.07 h	1.1 h (0.44 h / 0.03 h / 0.63 h)
	BTA15	2.45 h	40.16 h (0.21 h / 10.52 h / 29.43 h)	0.05 h	0.56 h (0.21 h / 0.02 h / 0.33 h)
	BTA25	1.37 h	28.30 h (0.11 h / 5.98 h / 22.21 h)	0.04 h	0.30 h (0.11 h / 0.01 h / 0.18 h)

The number of imputed SNP was 36 599, 20 145 and 10 981 for chromosomes 1, 15 and 25, respectively. Computing was performed on an Intel Xeon 2.13 Ghz processor.

^a^ The entire computing time for *MaCH* can be partitioned into three separate steps (in parentheses): pre-phasing of the reference population with *Beagle*, inference of tuning parameters based on 200 randomly selected animals of the validation population and actual genotype imputation with *MaCH.*

^b^*findhap*.*f90* was run exploiting the multi-threading option.

^c^ The entire computing time for *Minimac* can be partitioned into three separate steps (in parentheses): pre-phasing of the reference population with *Beagle*, pre-phasing of the validation population with *Beagle* and actual genotype imputation with *Minimac*.

**Table 5:** Evaluation of imputation accuracy

**Number of animals in reference and validation population**	***Beagle***			***MaCH***			***findhap*.*f90***			***Minimac***		
	**Correct alleles**	**Correct genotypes**^**a**^	**r**_**TG,IG**_	**Correct alleles**	**Correct genotypes**	**r**_**TG,IG**_	**Correct alleles**	**Correct genotypes**	**r**_**TG,IG**_	**Correct alleles**	**Correct genotypes**	**r**_**TG,IG**_
50 / 747	0.914	0.840	0.858	0.966	0.933	0.945	0.925	0.858	0.865	0.971	0.942	0.953
100 / 697	0.963	0.927	0.940	0.985	0.970	0.976	0.959	0.921	0.933	0.986	0.972	0.977
200 / 597	0.986	0.972	0.977	0.993	0.987	0.989	0.978	0.956	0.965	0.993	0.986	0.989
400 / 397	0.993	0.987	0.989	0.996	0.993	0.994	0.986	0.973	0.978	0.996	0.992	0.993

The mean allelic and genotypic accuracies over six chromosomes (BTA1, BTA5, BTA10, BTA15, BTA20, BTA25) were assessed for the imputed genotypes based on an increasing size of the reference population. Additionally, the correlation between true and imputed genotypes (r_TG,IG_) was calculated.

^a^ a genotype is correctly imputed if both alleles are correctly imputed.

The approach based on pre-phasing implemented in *Minimac* not only provided highly accurate imputed genotypes but was also computationally efficient (Table 4). However, *findhap*.*f90* was the most efficient computationally, especially for a large number of reference genotypes.

### Allelic imputation accuracy

**Figure 2:**
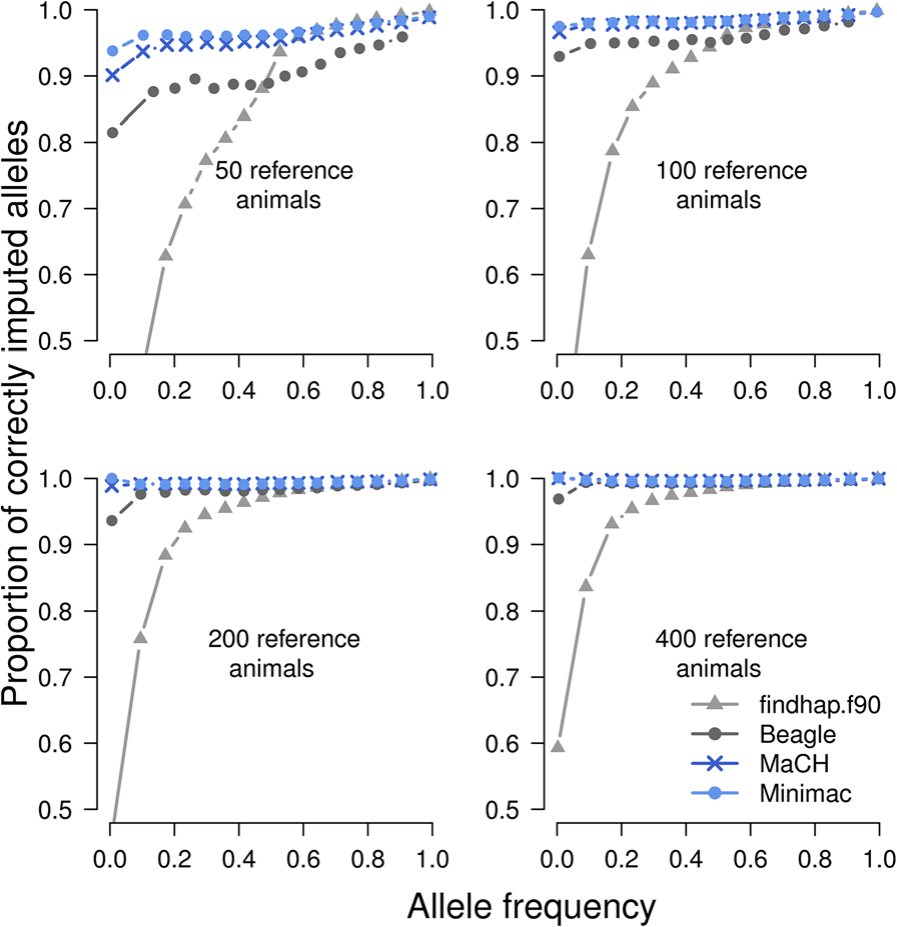
**Allelic imputation accuracy.** The proportion of correctly imputed alleles is displayed as a function of allele frequencies for *findhap*.*f90* (light grey), *Beagle* (dark grey), *MaCH* (blue) and *Minimac* (light blue) for an increasing reference population size. The curves were obtained by fitting a nonparametric local regression (LOESS).

The proportion of correctly imputed alleles was ~98.5% for *MaCH* and *Minimac*, for the scenario with 100 reference animals (Table 5). The corresponding r_TG,IG_ was > 0.975. *MaCH* and *Minimac* clearly outperformed *Beagle* and *findhap*.*f90* in all scenarios and provided the most accurate results for rare alleles. Imputation quality for rare alleles was very poor with *findhap*.*f90* (Figure 2). All algorithms inferred frequent alleles with high quality even when the number of reference animals was small.

### Individual imputation accuracy

**Figure 3:**
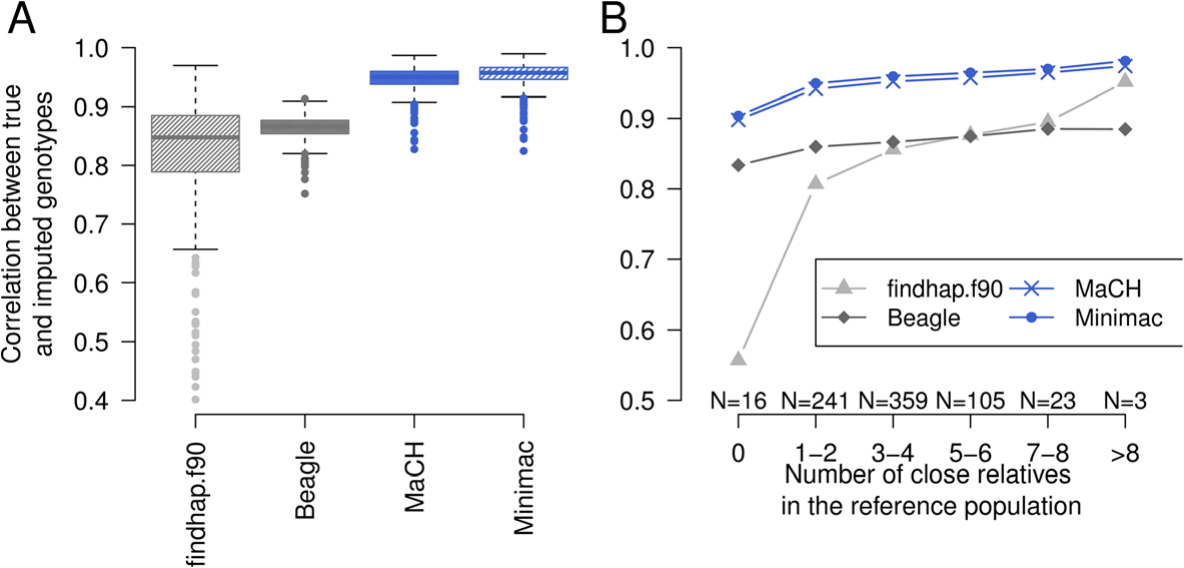
**Individual imputation accuracy for the scenario with 50 reference animals.** Barplots indicate the correlation between true and imputed genotypes (r_TG,IG_) for 747 animals based on 50 reference animals (**A**). The individual r_TG,IG_ increased considerably as the number of close relatives increased (coefficient of relationship >0.12) in the reference population (**B**).

The quality of the imputed genotypes varied considerably between animals (Figure 3A). The extent of genotype information from relatives in the reference population was the major determinant of the individual imputation accuracy. For the scenario with 50 reference animals, most validation animals (n = 621) had no first-degree relatives in the reference population (Table 1). We considered that reference and validation animals are close relatives if their relationship was above 0.12. While r_TG,IG_ was < 0.90 for all algorithms without including high-density genotype information from closely related animals, the imputation accuracy increased considerably as the number of relatives in the reference population increased (Figure 3B). *MaCH* and *Minimac* provided highly accurate genotypes, even if the number of relatives in the reference population was small. With *MaCH* and *Minimac*, the average r_TG,IG_ exceeded 0.94 for validation animals which had at least one related animal with r ≥ 0.12 in the reference population. The accuracy of *findhap*.*f90* increased considerably as the number of relatives in the reference population increased. With *Beagle*, r_TG,IG_ never exceeded 0.885 for the scenario with 50 reference animals.

The impact of genotype information from relatives decreased as the size of the reference population increased when applying the population-based imputation tools *Beagle*, *MaCH* and *Minimac*. In contrast, the quality of the imputed genotypes obtained with *findhap*.*f90* increased considerably as the number of relatives increased, even for the scenario with 400 reference animals (see Additional file 4).

### Imputation accuracy obtained based on randomly selected reference animals

Ten subsets of 50 randomly selected animals that explained between 68.9% and 71.9% of the gene pool of the entire data set were used as reference populations to impute genotypes for 747 validation animals on chromosome 20. The imputation accuracy obtained based on these randomly selected reference animals was lower compared with r_TG,IG_ obtained using the most informative animals as reference individuals across all replications for the four imputation tools (Table 6). Using the most informative instead of random animals as reference population increased r_TG,IG_ especially for *findhap*.*f90* (0.876 *vs*. 0.837). However, *Minimac* and *MaCH* provided high imputation accuracy even with randomly selected reference populations.

**Table 6:** Imputation accuracy on chromosome 20 based on varying reference populations

	**50 most informative animals**	**50 randomly selected animals**		
		**Mean**	**Min**	**Max**
*Beagle*	0.866	0.854	0.841	0.864
*MaCH*	0.949	0.942	0.937	0.946
*findhap*.*f90*	0.876	0.837	0.812	0.856
*Minimac*	0.957	0.947	0.943	0.951

The correlation between true and imputed genotypes (r_TG,IG_) based on the 50 most informative animals as reference population is compared with r_TG,IG_ obtained with 50 randomly selected reference animals. The mean, minimum and maximum r_TG,IG_ obtained with randomly selected reference animals are displayed across ten replications for the four imputation tools.

### Identification of misplaced SNP

**Figure 4:**
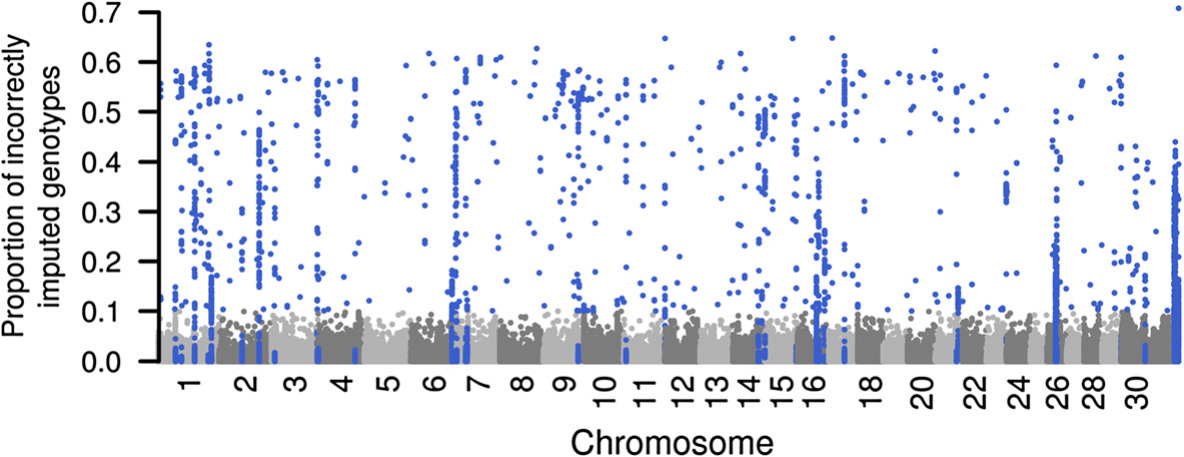
**Genome**-**wide distribution of the proportion of correctly imputed genotypes.** Genotypes of 599 535 SNP were imputed for 397 animals based on haplotype information of 400 reference animals using *Minimac*. Blue dots represent 5039 SNP within regions of poor imputation quality probably representing misplaced SNP.

*Minimac* was used for genome-wide imputation of high-density genotypes. Of 639 214 SNP, 39 679 SNP were retained for 397 validation animals, while genotypes for the remaining 599 535 SNP were imputed using 400 reference animals. With this design r_TG,IG_ was equal to 0.993 across six analysed chromosomes (Table 5). The genome-wide distribution of the imputation accuracy revealed genomic regions with poor imputation quality (Figure 4). Analysis of these regions showed that misplacement of SNP increased the fraction of poorly imputed genotypes. Poor imputation quality along entire chromosomal segments results from intra-chromosomal misplacement of adjacent SNP. However, analysis of LD also indicated inter-chromosomal misplacement of individual SNP (see Additional file 5). A total of 5039 out of 599 535 SNP (0.84%) was identified as probably misplaced using this procedure.

## Discussion

Four imputation tools were evaluated using a data set consisting of 797 bulls of the German FV population genotyped at 639 214 SNP. The reference animals were selected to capture the greatest proportion of the gene pool of the genotyped population. Using *Minimac*, up to 97.1% of the alleles were correctly imputed based on 50 pre-selected reference animals. Imputation accuracy based on genotypes of randomly selected reference animals was slightly, albeit consistently lower (Table 6). Brøndum et al. [31] used *Beagle* to impute high-density genotypes in three cattle breeds based on ~200 reference animals and obtained r_TG,IG_ ranging from 0.925 to 0.973. In our study, *Beagle* yielded an r_TG,IG_ of 0.977 with 200 pre-selected reference animals. In the Holstein-Friesian breed, Erbe et al. [32] obtained 97.7% of correctly imputed genotypes with *Beagle* using ~400 randomly selected reference animals. In our study, using *Beagle* with 400 pre-selected animals as reference population yielded 98.7% of correctly imputed genotypes. However, *MaCH* and *Minimac* yielded the same imputation accuracy with 200 reference animals only. Selecting highly informative reference animals (*i*.*e*. key animals) maximises the proportion of genes/haplotypes in the validation population that can be traced back to these key animals and thus maximises imputation accuracy while minimizing genotyping costs [20,33,34]. Our findings demonstrate that pre-selecting highly-informative reference animals is slightly beneficial for subsequent genotype imputation. The most influential animals have been identified in various cattle populations [35,36] and such 'key animals' will be used for whole-genome re-sequencing. Simulations have shown that imputation of sequence information from a restricted number of highly informative individuals is feasible [21]. Genotyping a large number of animals at high-density and subsequently imputing the whole-genome sequence information from a small number of carefully selected 'key animals' might lead to even higher accuracy, since imputation quality strongly depends on the marker density in both reference and validation populations [34,37,38]. However, our findings also show that the choice of a suitable imputation algorithm is more crucial than the selection of 'key animals' to obtain high imputation accuracy based on a small number of reference animals.

Imputation accuracy increased as the size of the reference population increased, which agrees with [17,37,39]. However, the performance of imputation tools varied considerably, especially when the number of animals and relatives with high-density genotypes was limited. *MaCH* and *Minimac* provided highly accurate imputed genotypes, even with only 50 reference animals compared to *Beagle* and *findhap*.*f90*. This advantage of *MaCH* and *Minimac* for genotype imputation based on a small reference population agrees with the reports of Browning and Browning [40] and Pei et al. [27]. If the size of the reference population increases, the accuracies of the imputation tools converge, which agrees with findings of Browning and Browning [13]. For the scenarios with 50 and 100 reference animals, the approach based on pre-phasing and implemented with *Minimac* provided the most accurate genotypes. *Minimac* was run after phasing both reference and validation populations with *Beagle*, disregarding pedigree information. Accounting for pedigree information might further improve the quality of phasing and thus the accuracy of subsequent genotype imputation [15,16,30]. In our study, the number of validation animals with close relatives in the reference population was very small. Thus, we found no increase in imputation accuracy with *findhap*.*f90*. However, if the number of closely related reference animals is increased, imputation algorithms using both pedigree and population information are likely to outperform tools using population information only [41]. The pre-phasing approach applied in the present study is preferable when the number of related reference animals is small. Besides allowing for a high imputation accuracy, imputation approaches based on pre-phasing are computationally efficient. The reference genotypes need to be phased only once and the phasing step can be separated from the actual imputation step [26,42]. This restricts the computational burden of genotype imputation in routine implementations such as genomic prediction. Previous studies have shown that long-range phasing and haplotype library imputation provide accurately imputed genotypes in livestock populations at a low computational input when the reference population is large [6,43-45]. Our results indicate that pre-phasing might slightly increase imputation accuracy, particularly when the number of reference genotypes is limited. The benefit of pre-phasing is expected to result from capturing LD effects at a better resolution [26]. Thus, pre-phasing based approaches might become the method of choice to impute the entire sequence information based on the re-sequencing of a limited number of key genomes in livestock populations.

Two population-based approaches that exploit LD without explicitly considering pedigree information (*MaCH*, *Minimac*) outperformed *findhap*.*f90* that takes relationships into account. *findhap*.*f90* was specifically designed to impute genotypes using large data sets and exploiting comprehensive pedigree information [6]. In contrast, our data set comprised 797 animals only, mainly born between 1997 and 2004. Furthermore, the number of genotyped relatives in the reference population was very small for most of the animals in the validation population, resulting in comparably low overall imputation accuracy when using *findhap*.*f90*. However, imputation with *findhap*.*f90* provided > 98% of correctly imputed genotypes when a substantial number of the relatives with genotypes was present in the reference population (see Additional file 4). This agrees with findings for the American Holstein-Friesian population [6]. However, comparing imputation accuracy across studies and breeds is difficult since data sets and population-specific parameters (*e*.*g*. LD, effective population size (N_e_)) might differ substantially. While recent N_e_ estimates for the Holstein-Friesian population range from < 100 to 114 [35,46,47], N_e_ for the FV population is considerably higher (see Additional file 6). Low LD, which is typical for populations with large N_e_[48], complicates genotype imputation considerably [27]. However, in populations with small N_e_ (*e*.*g*. Jersey cattle [35]), genotype imputation based on a small number of carefully selected reference animals yields a reasonable accuracy [32]. Our results demonstrate that genotyping at least 100 pre-selected animals at high density and subsequently applying population-based imputation yielded highly reliable genotypes for the analysed subset of the FV population, although N_e_ is comparatively large. However, the animals in our study are highly selected artificial insemination bulls and might not fully reflect the haplotype diversity of the entire FV population.

Genome-wide analysis of imputation accuracy also allowed misplaced SNP to be identified. Although misplaced SNP are particularly obstructive for haplotype-based analyses (*e*.*g*. identification of selective sweeps) [49], the position of significantly associated SNP in genome-wide association studies should also be validated to avoid misinterpretations. Analysis of genomic regions with poor imputation quality revealed 5039 SNP that are most likely misplaced. Recently, Erbe et al. [32] showed similar results. The total number of misplaced SNP might be even higher, since SNP with very low MAF (< 1%) were excluded for the evaluation of imputation accuracy. Furthermore, our procedure is not suitable to reveal misplaced SNP within short distances. However, the proportion of misplaced SNP in the high-density array used here is slightly higher than in the BovineSNP50 Bead chip [50], which is most likely due to a better resolution of the high-density marker map. The number of misplaced SNP detected here is considerably higher than reported by Fadista and Bendixen [51], who relied on a more precise assembly of the reference sequence. However, LD-based procedures make it possible to realign SNP positions despite imperfectly assembled reference genomes.

## Conclusions

Genotype imputation allows different marker panels to be combined and missing genotypes to be infered *in silico*. The quality of the imputed genotypes strongly depends on the amount of genotype information that is available from relatives. However, population-based imputation tools provide highly-reliable genotypes even if the number of reference animals is small. In addition, imputation accuracy increases if the animals of the reference panel are chosen to maximally contribute to the gene pool of the imputation population. Pre-phasing the genotypes of both the reference and validation populations not only results in highly accurately imputed genotypes but is also computationally efficient.

## Abbreviations

FV: Fleckvieh; HMM: Hidden Markov model; LD: Linkage disequilibrium; MAF: Minor allele frequency; Ne: Effective population size; QTN: Quantitative trait nucleotide; SNP: Single nucleotide polymorphism.

## Competing interests

The authors declare that they have no competing interests.

## Authors’ contributions

HP and RF conceived and designed the experiments, HP and BA performed the experiments, RE, CE and KUG contributed pedigree and genotype data and HP and RF wrote the manuscript. All authors have read and approved the final manuscript.

## Supplementary Material

Additional file 1 **Birth years of 814 genotyped bulls of the Fleckvieh breed.** Birth years ranged from 1970 to 2007 with 90.2% of the animals born between 1997 and 2004. Click here for file

Additional file 2 **Pairwise pedigree *vs*. genomic relationship.** Pairwise pedigree *vs*. genomic relationship for 806 Fleckvieh bulls passing stringent quality before (A) and after (B) the exclusion of nine animals with inconsistencies. Click here for file

Additional file 3 Imputation pipelines for the four different imputation tools. Click here for file

Additional file 4 **Individual imputation accuracy for the scenario with 400 reference animals.** The individual imputation accuracy (r_TG,IG_) increased only slightly with an increasing number of second-degree relatives in the reference population for *Beagle*, *MaCH* and *Minimac*. However, a strong increase in accuracy was observed for *findhap*.*f90*. Click here for file

Additional file 5 **Identification of misplaced SNP on chromosome 26.** The distribution of the proportion of imputation errors highlights the regions with poor imputation quality on chromosome 26 (A). Blue and red symbols indicate 391 SNP that were considered as misplaced. The red symbol indicates BovineHD2600003844, which is located on BTA26 (according to the UMD3 assembly). However, analysis of linkage disequilibrium with all other SNP indicates that the proximal region of BTA11 is the actual position (B). The pairwise linkage disequilibrium on BTA26 is shown as a function of the pairwise distance before (C) and after (D) the exclusion of 391 probably misplaced SNP (r^2^-values below 0.1 are omitted). Click here for file

Additional file 6 Estimation of the effective population size for the Fleckvieh population. Click here for file
