# Supplementary figures and images for: Imputation of high-density genotypes in the Fleckvieh cattle population
Source: Genet Sel Evol. 2013 Feb 13;45(1):3. doi: 10.1186/1297-9686-45-3 (PMC3598996; doi:10.1186/1297-9686-45-3)

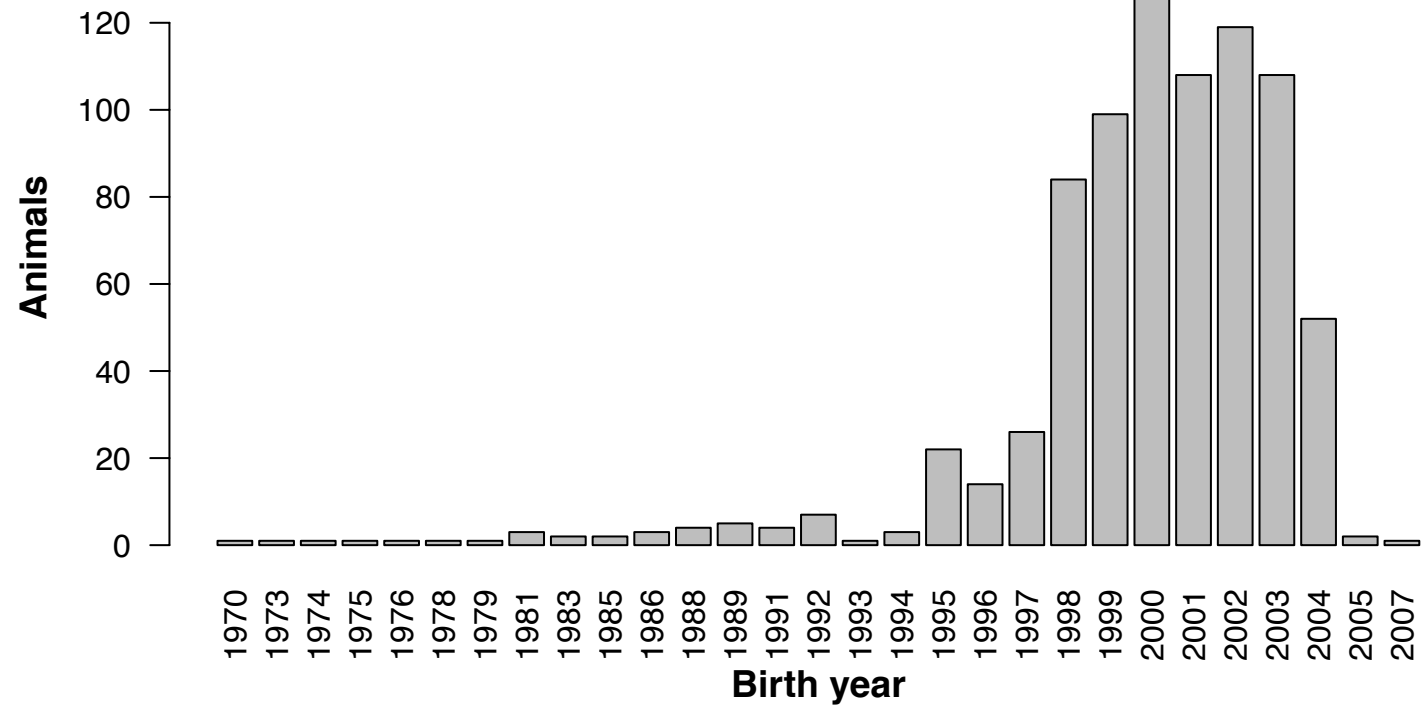

Supplement: Additional file 1 — Birth years of 814 genotyped bulls of the Fleckvieh breed. Birth years ranged from 1970 to 2007 with 90.2% of the animals born between 1997 and 2004. [file 1297-9686-45-3-S1.pdf]

**A**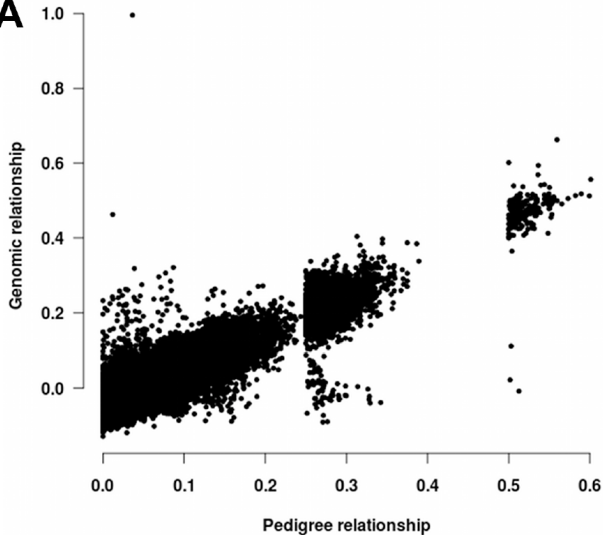**B**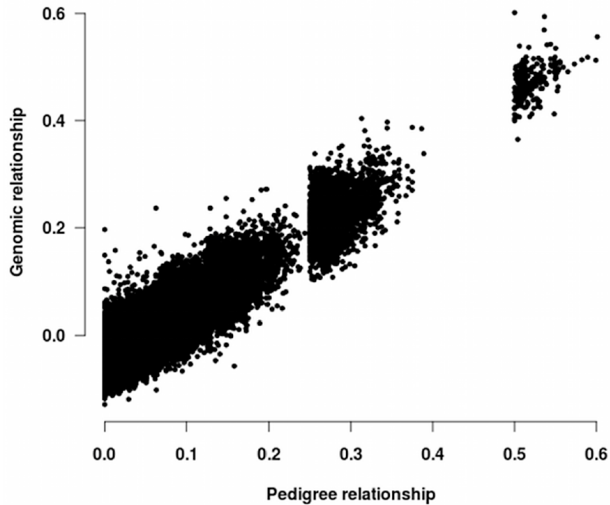

Supplement: Additional file 2 — Pairwise pedigree vs. genomic relationship. Pairwise pedigree vs. genomic relationship for 806 Fleckvieh bulls passing stringent quality before (A) and after (B) the exclusion of nine animals with inconsistencies. [file 1297-9686-45-3-S2.pdf]

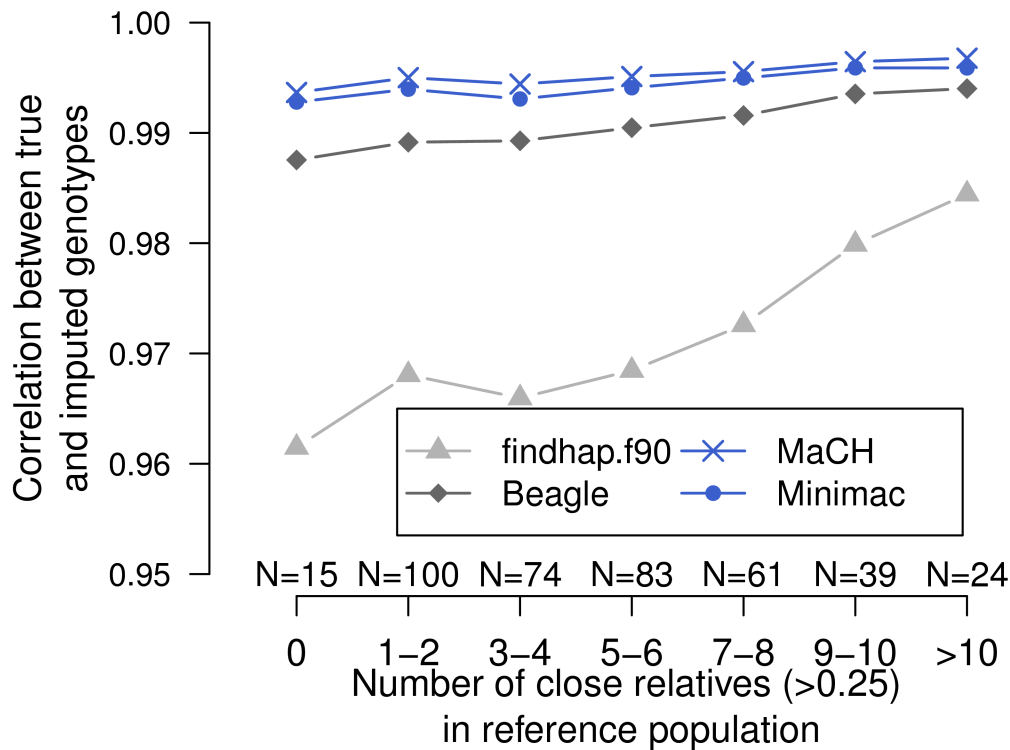

Supplement: Additional file 4 — Individual imputation accuracy for the scenario with 400 reference animals. The individual imputation accuracy (rTG,IG) increased only slightly with an increasing number of second-degree relatives in the reference population for Beagle, MaCH and Minimac. However, a strong increase in accuracy was observed for findhap.f90. [file 1297-9686-45-3-S4.pdf]

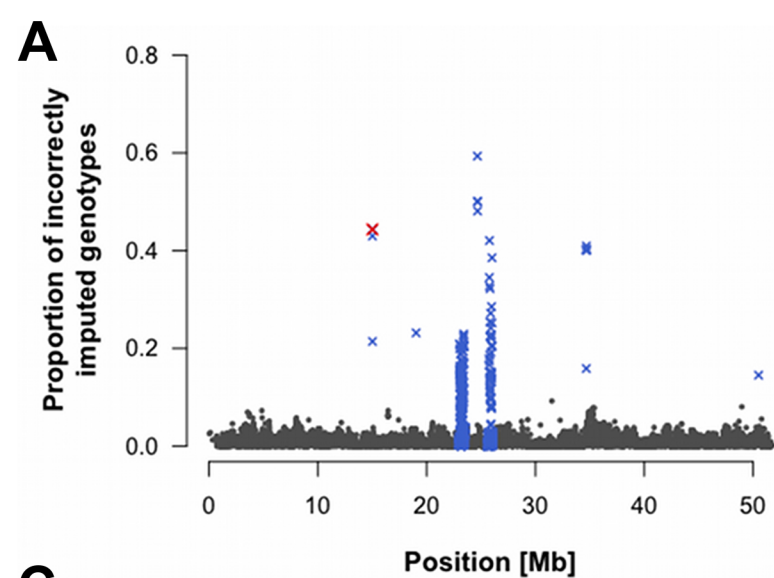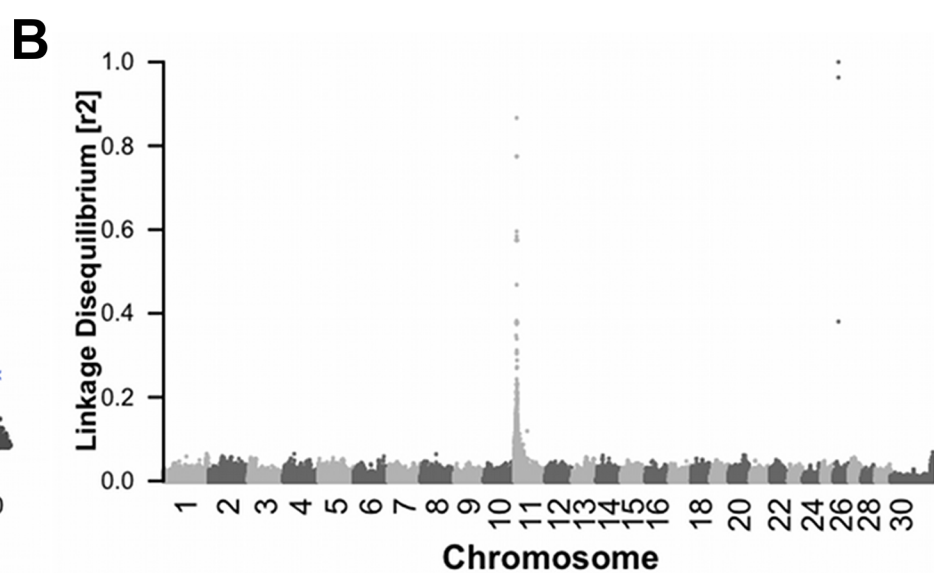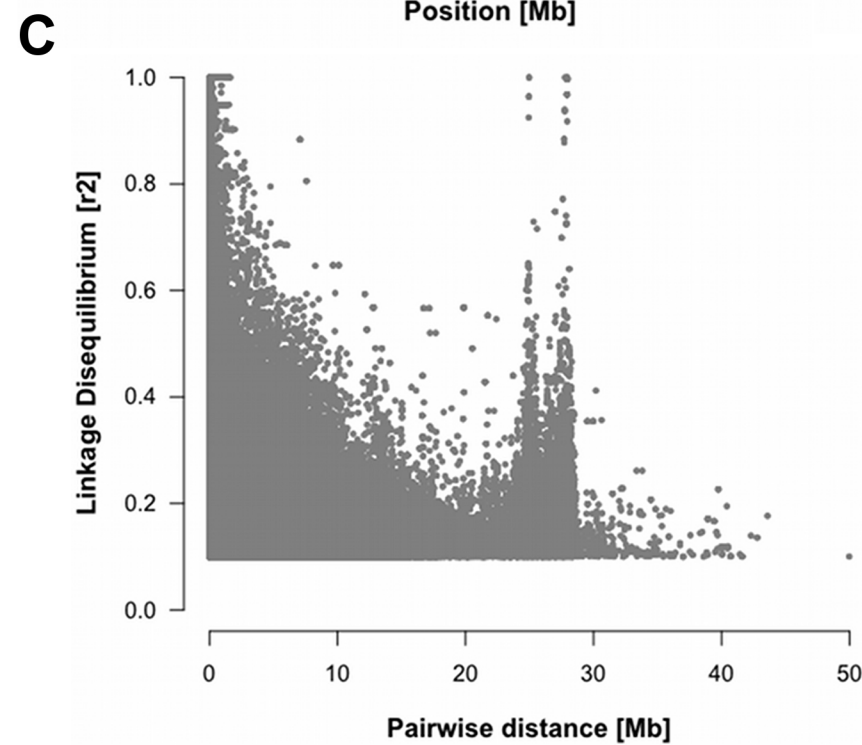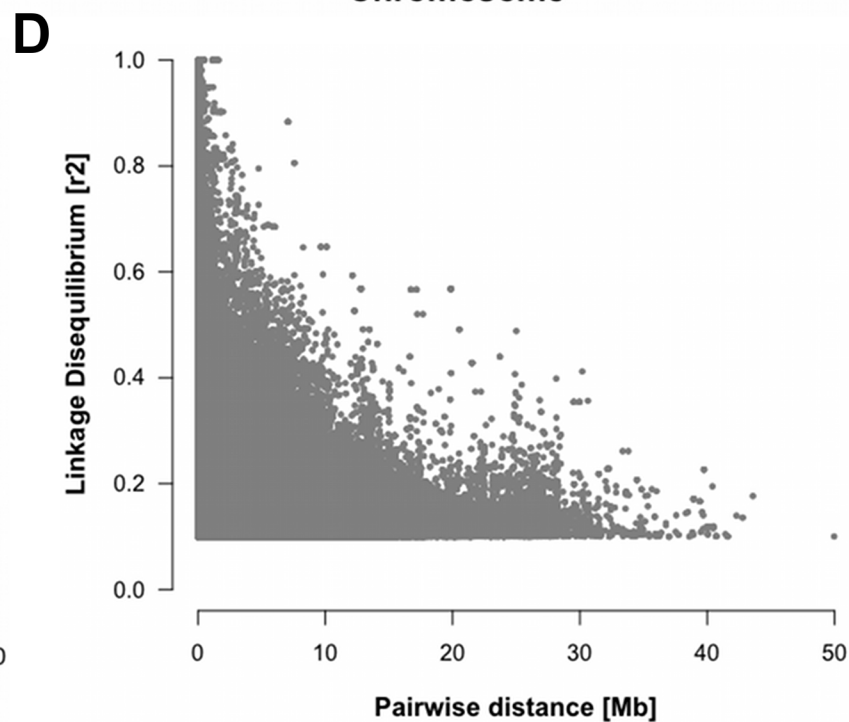

Supplement: Additional file 5 — Identification of misplaced SNP on chromosome 26. The distribution of the proportion of imputation errors highlights the regions with poor imputation quality on chromosome 26 (A). Blue and red symbols indicate 391 SNP that were considered as misplaced. The red symbol indicates BovineHD2600003844, which is located on BTA26 (according to the UMD3 assembly). However, analysis of linkage disequilibrium with all other SNP indicates that the proximal region of BTA11 is the actual position (B). The pairwise linkage disequilibrium on BTA26 is shown as a function of the pairwise distance before (C) and after (D) the exclusion of 391 probably misplaced SNP (r2-values below 0.1 are omitted). [file 1297-9686-45-3-S5.pdf]
