# Supplementary material for: Imputation of high-density genotypes in the Fleckvieh cattle population
Source: Genet Sel Evol. 2013 Feb 13;45(1):3. doi: 10.1186/1297-9686-45-3 (PMC3598996; doi:10.1186/1297-9686-45-3)
Supplement: Additional file 3 — Imputation pipelines for the four different imputation tools. [file 1297-9686-45-3-S3.pdf]

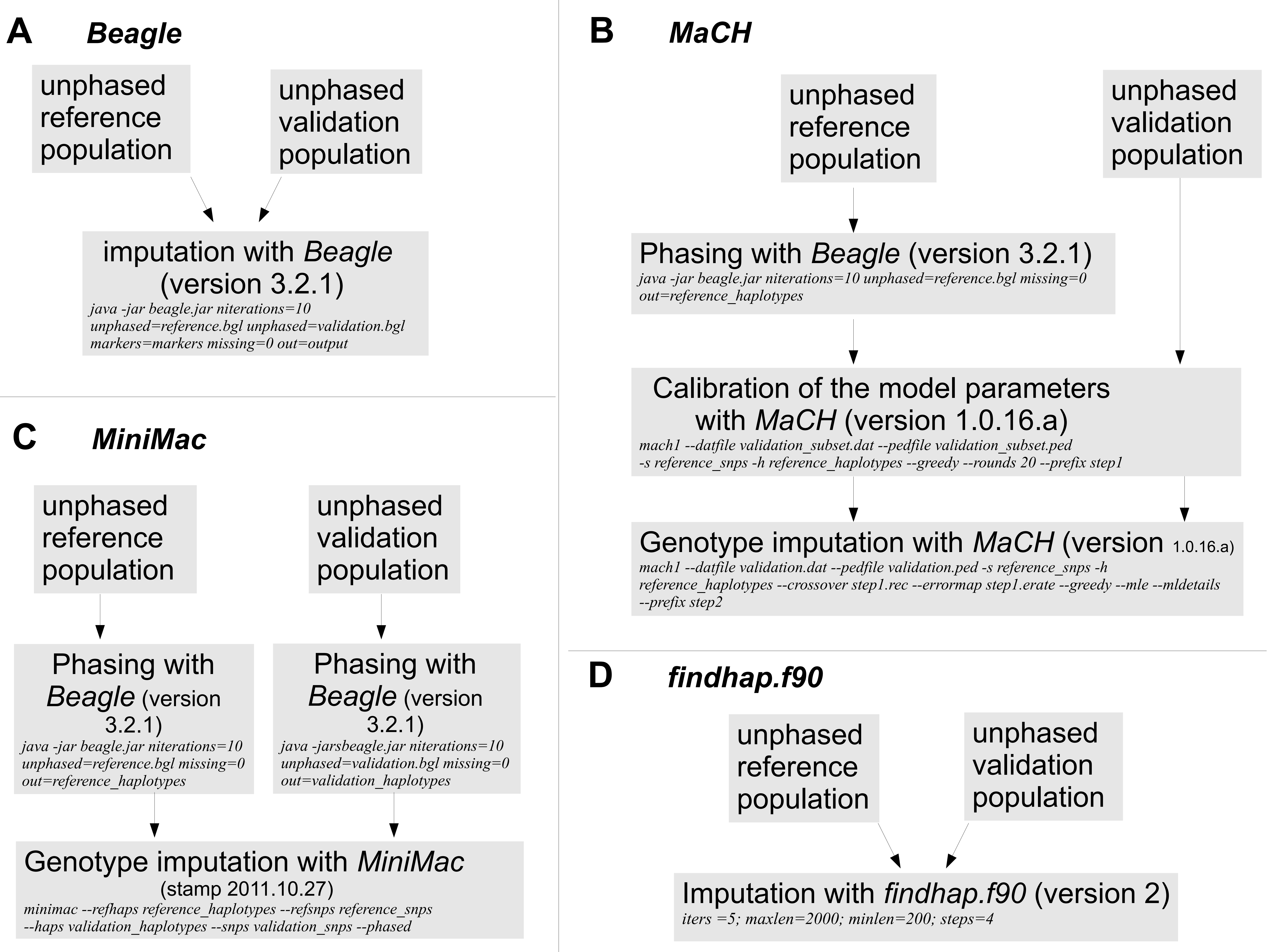
A
Beagle
unphased reference population
unphased validation population
imputation with Beagle (version 3.2.1)
java -jar beagle.jar niterations=10 unphased=reference.bgl unphased=validation.bgl markers=markers missing=0 out=output
B
MaCH
unphased reference population
unphased validation population
Phasing with Beagle (version 3.2.1)
java -jar beagle.jar niterations=10 unphased=reference.bgl missing=0 out=reference_haplotypes
Calibration of the model parameters with MaCH (version 1.0.16.a)
mach1 --datfile validation_subset.dat --pedfile validation_subset.ped -s reference_snps -h reference_haplotypes --greedy --rounds 20 --prefix step1
Genotype imputation with MaCH (version 1.0.16.a)
mach1 --datfile validation.dat --pedfile validation.ped -s reference_snps -h reference_haplotypes --crossover step1.rec --errormap step1.erate --greedy --mle --mldetails --prefix step2
C
MiniMac
unphased reference population
unphased validation population
Phasing with Beagle (version 3.2.1)
java -jar beagle.jar niterations=10 unphased=reference.bgl missing=0 out=reference_haplotypes
Phasing with Beagle (version 3.2.1)
java -jarsbeagle.jar niterations=10 unphased=validation.bgl missing=0 out=validation_haplotypes
Genotype imputation with MiniMac (stamp 2011.10.27)
minimac --refhaps reference_haplotypes --refsnps reference_snps --haps validation_haplotypes --snps validation_snps --phased
D
findhap.f90
unphased reference population
unphased validation population
Imputation with findhap.f90 (version 2)
iters =5; maxlen=2000; minlen=200; steps=4
