# Supplementary material for: Imputation of high-density genotypes in the Fleckvieh cattle population
Source: Genet Sel Evol. 2013 Feb 13;45(1):3. doi: 10.1186/1297-9686-45-3 (PMC3598996; doi:10.1186/1297-9686-45-3)
Supplement: Additional file 6 — Estimation of the effective population size for the Fleckvieh population. [file 1297-9686-45-3-S6.pdf]

ADDITIONAL FILE 6

Estimation of the effective population size in the Fleckvieh population

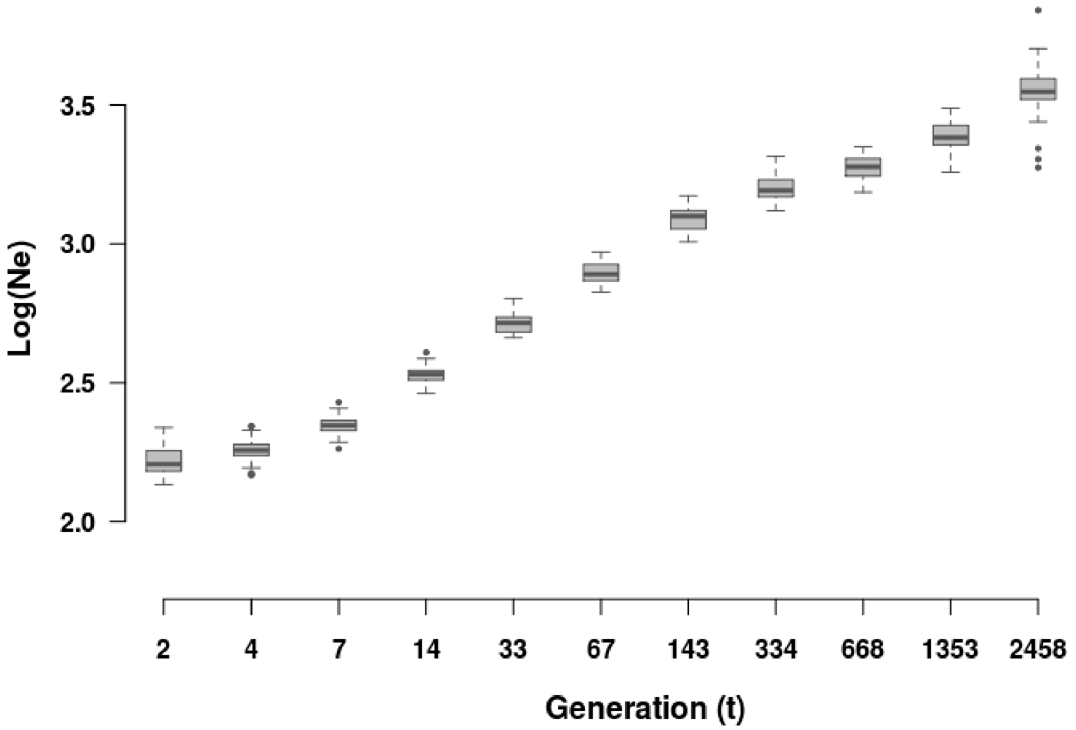

A maximum effective population size (Ne) of 3529 was estimated at ~2500 generations ago. However, the Ne decreased considerably within the last generations. While the past Ne was estimated to ~340, ~222 and ~181 for 14, 7 and 4 generations ago, respectively, the past Ne ~2 generations ago was estimated to 161 (±22.8) only. Individual estimates for the 29 autosomes range from 136 (BTA14) to 218 (BTA29) for ~2 generations ago.

The past effective population size (N_e) was estimated based on a comprehensive dataset comprising 2842 FV bulls born between 1998 and 2005 genotyped with the Illumina BovineSNP 50K Bead chip. After stringent quality control genotypes for 42,237 autosomal SNPs were considered. The marker-based N_e was estimated using $r^2 = \frac{1}{4 N_e c + 1} + e$ ((Sved 1971),(Tenesa et al. 2007)), where r^2 is the pairwise linkage disequilibrium and c is the genetic distance. Physical distances were converted to genetic distances (Arias et al. 2009) under the assumption of constant recombination rates. N_e at generation t was estimated for all autosomes separately within different marker distance bins (<0.025, 0.025-0.050, 0.050-0.1, 0.1-0.2, 0.2-0.5, 0.5-1, 1-2, 2-5, 5-10, 10-15, 15-30 cM) using non-linear least squares as implemented in the R function *nls*(). The generation t was calculated as t = 1/2c, where c was averaged across all corresponding pairwise marker distances.

Arias, J.A. et al., 2009. A high density linkage map of the bovine genome. *BMC Genetics*, 10, p.18.

Sved, J.A., 1971. Linkage disequilibrium and homozygosity of chromosome segments in finite populations. *Theoretical Population Biology*, 2(2), pp.125-141.

Tenesa, A. et al., 2007. Recent human effective population size estimated from linkage disequilibrium. *Genome Research*, 17(4), pp.520-526.
